# Integrated Source of Path-Entangled Photon Pairs with Efficient Pump Self-Rejection

**DOI:** 10.3390/nano10101952

**Published:** 2020-09-30

**Authors:** Pablo de la Hoz, Anton Sakovich, Alexander Mikhalychev, Matthew Thornton, Natalia Korolkova, Dmitri Mogilevtsev

**Affiliations:** 1 School of Physics and Astronomy, University of St Andrews, North Haugh, St Andrews KY16 9SS, UK; mt45@st-andrews.ac.uk (M.T.); nvk@st-andrews.ac.uk (N.K.); 2 B. I. Stepanov Institute of Physics, National Academy of Sciences of Belarus, Nezavisimosti Ave. 68-2, 220072 Minsk, Belarus; sakovich.2.718281828459045@gmail.com (A.S.); mikhalychev@gmail.com (A.M.); d.mogilevtsev@ifanbel.bas-net.by (D.M.)

**Keywords:** four-wave mixing, coherent diffusive photonics, entangled photons generation

## Abstract

We present a theoretical proposal for an integrated four-wave mixing source of narrow-band path-entangled photon pairs with efficient spatial pump self-rejection. The scheme is based on correlated loss in a system of waveguides in Kerr nonlinear media. We calculate that this setup gives the possibility for upwards of 100 dB pump rejection, without additional filtering. The effect is reached by driving the symmetric collective mode that is strongly attenuated by an engineered dissipation, while photon pairs are born in the antisymmetric mode. A similar set-up can additionally be realized for the generation of two-photon NOON states, also with pump self-rejection. We discuss the implementation of the scheme by means of the coherent diffusive photonics, and demostrate its feasibility in both glass (such as fused silica-glass and IG2) and planar semiconductor waveguide structures in indium phosphide (InP) and in silicon.

## 1. Introduction

The generation of photon pairs is a staple tool of modern quantum technologies. Twin photons have found far-reaching applications in a wide range of fields, from quantum communications to imaging, metrology, and LIDARs [1,2,3]. One of the established methods for producing photon pairs is the spontaneous four-wave mixing (SFWM) process in Kerr-nonlinear structures [4,5,6]. This method is very promising, with the perspective to create integrated sources of photon pairs that are compatible with the other photonic blocks necessary, for example, for quantum processors or quantum key distribution systems [7,8]. Such a process can be realized in integrated waveguiding structures (for example, in silicon or indium phospide (InP) platforms), which render them very suitable for building quantum photonic circuits [9,10,11,12].

One of the main problems in implementing SFWM for photon pair generation is pump rejection, especially with CW pumping. Because, in the SFWM process, two photons are converted into signal and idler photons, and all four are of close frequencies, achieving large pump rejection can be challenging and it requires quite exquisite filtering [6]. This is often accomplished via filtering setups far larger in size that the nonlinear device producing photon pairs. Recently, there has been great interest in producing on-chip filters that reject the pump [9,12]. Generally, the majority of these filtering schemes are based on very precise frequency filtering allowing for transmission bands less than 1 nm wide and achieving more than 100 dB transmission of pass-band to stop-band contrast [9,12,13,14]. Only recently, a theoretical proposal using photonic crystals has appeared suggesting to exploit spatial features, i.e., suppress coupling of the output mode to the pump by symmetry considerations [15]. Similar spatial filtering ideas were also suggested for the integrated source of photon pairs based on the three-wave mixing by the spontaneous parametric down-conversion (SPDC) [16,17].

**Figure 1:**
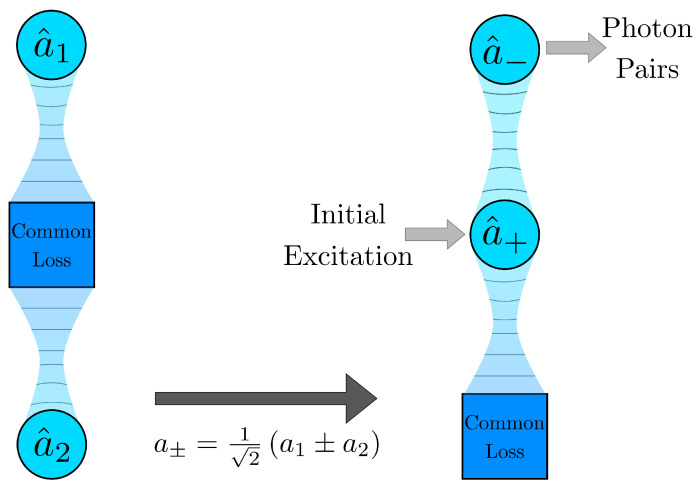
The scheme of the photon-pair generator with in-built pump rejection on the basis of two single-mode waveguides coupled to the common loss reservoir.

In this work, we suggest a novel and simple way for realizing efficient on-chip pump rejection by the SFWM process in a waveguide structure with engineered loss. In essence, this structure consists of two waveguides coupled only dissipatively through a common reservoir, as in Figure 1. Coupling to the common reservoir defines a superposition mode subject to a strong engineered loss. If one only pumps this modal superposition, photon pairs are born in the orthogonal mode and they travel through the waveguides, whereas the pump exponentially decays along the waveguide. Our scheme allows for the pump to be filtered out, even if the pump, signal, and idler are of the same frequency.

Such a “built-in” filtering can be further enhanced by exploiting the symmetry properties of the modes (as it was done in recent works with SPDC [16,17]): the output from both waveguides can be interfered on a beamsplitter, in order to filter out the possible remnants of the symmetric mode and put both photons in the same spatial mode.

Furthermore, our pump-rejecting scheme can be modified in order to produce a pair of photons in two collective modes (i.e., in four spatial modes), which can then be used to produce two-photon NOON states [18,19]. These are multi-photon entangled states corresponding to the superposition of N=2 photons in the first mode with zero photons in the second mode, and vice versa.

Note, that systems containing Kerr-type nonlinearities have already been considered as a source of entangled states [20,21,22]. Systems of two coupled waveguides with Kerr nonlinear medium used in one or both channels have been studied in the context of co- and contra-directional couplers (for review, see [23]). Quantum statistics and dynamics of Kerr nonlinear couplers with linear and nonlinear coupling, including regimes of varying linear coupling, have been attracting interest for more than three decades now and shown to lead to the whole range of different non-trivial quantum effects, such as collapses and revivals of oscillations, sub-Poissonian photon statistics in single and in compound modes [24,25], as well as higher-order nonclassicalities: quantum entanglement, squeezing, and antibunching [26]. Already, the possibility to achieve novel regimes that emulate the dynamics of complex many-body systems has been investigated (see, e.g., [27]). Nowadays, a whole new active field of research has emerged, topological photonics [28], where also our dissipatively coupled systems may contribute. The principle difference of the systems that is presented in this paper to the nonlinear directional couplers is the use of the engineered non-linear loss as the main mechanism determining the device functionality [29,30,31].

## 2. Scheme

Let us introduce the simplest model to describe the *modus operandi* of our generator with in-built pump rejection.

The basic scheme that we suggest for the pair generation is just two identical, self-Kerr nonlinear single-mode waveguides solely coupled by common loss in a symmetric way (Figure 1). The mode dynamics in this device can be described by the following master equation for the density matrix ρ:

(1) $$\begin{array}{c} \frac{d}{dt}\rho =-i\frac{U}{2}\left[{\left(a_{1}^{†}\right)}^{2}a_{1}^{2}+{\left(a_{2}^{†}\right)}^{2}a_{2}^{2},\rho \right]+\frac{1}{2}\Gamma L\left(a_{1}+a_{2}\right)\rho +\gamma \left(L\left(a_{1}\right)+L\left(a_{2}\right)\right)\rho , \end{array}$$

where $a_{j}$, $a_{j}^{†}$ are bosonic annihilation and creation operators of *j*-th mode, *U* is the nonlinearity, γ and Γ are individual and collective loss rates, and the dissipator is $L\left(a_{j}\right)\rho =a_{j}\rho a_{j}^{†}-\frac{1}{2}\rho a_{j}^{†}a_{j}-\frac{1}{2}a_{j}^{†}a_{j}\rho$.

After transforming to the basis $a_{\pm }=\frac{1}{\sqrt{2}}\left(a_{1}\pm a_{2}\right)$, Equation (1) becomes

(2) $$\begin{array}{c} \frac{d}{dt}\bar{\rho }=-i\left[H+V,\bar{\rho }\right]+\left(\Gamma +\gamma \right)L\left(a_{+}\right)\bar{\rho }+\gamma L\left(a_{-}\right)\bar{\rho }, \end{array}$$

where the Kerr interaction Hamiltonian is

(3) $$\begin{array}{c} H=\frac{U}{4}\left(n_{+}^{2}+n_{-}^{2}+4n_{+}n_{-}-n_{+}-n_{-}\right), \end{array}$$

and the two-photon exchange Hamiltonian is

(4) $$\begin{array}{c} V=\frac{U}{4}\left({\left(a_{+}^{†}\right)}^{2}a_{-}^{2}+h.c.\right). \end{array}$$

the operators $n_{\pm }=a_{\pm }^{†}a_{\pm }$ are photon-number operators for the superposition modes.

The scheme of two self-Kerr nonlinear modes coupled by the common loss was already considered in a number of works [29,30,31,32]. However, it was mostly considered as a way to engineer two-photon loss for generating non-classical states from the initial classical input. Collective loss was assumed to be strong, and the symmetric mode $a_{+}$ was usually adiabatically eliminated.

Here, we exploit a different and somewhat counter-intuitive strategy. Let us initially excite only the symmetric mode $a_{+}$. In realistic waveguiding structures, Kerr nonlinearity is small. Hence, if, initially, the symmetric mode is in a coherent state and $U\langle n_{+}\rangle \ll \Gamma$, both the Kerr nonlinearity and interaction with the antisymmetric mode will hardly affect the symmetric mode. Its state will remain coherent and uncorrelated with the state of the antisymmetric mode.

Under this assumption, after averaging over the states of the symmetric mode, Equation (2) becomes the following equation for the single-mode density matrix ϱ:

(5) $$\begin{array}{c} \frac{d}{dt}\varrho \approx -i\left[H_{-}+V_{-}\left(t\right),\varrho \right]+\gamma L\left(a_{-}\right)\varrho , \end{array}$$

where the driving-independent part is $H_{-}=\frac{U}{4}\left(n_{-}^{2}-n_{-}\right)$. The driving term reads

(6) $$\begin{array}{c} V_{-}\left(t\right)=U{\left|\alpha_{+}\left(t\right)\right|}^{2}n_{-}+\frac{U}{4}\left(\alpha^{2}\left(t\right){\left(a_{-}^{†}\right)}^{2}+h.c.\right), \end{array}$$

with

(7) $$\begin{array}{c} \alpha_{+}\left(t\right)\approx \alpha_{+}\left(0\right)\exp\left\{-\frac{1}{2}\left(\Gamma +\gamma \right)t\right\}, \end{array}$$

where $\alpha_{+}\left(0\right)$ is the initial amplitude of the symmetric mode.

For simplicity sake, let us first assume that the waveguide loss is negligibly small, γT≪1, where *T* is the total propagation time, and the driving is not very strong, $U|\alpha_{+}{\left(0\right)|}^{2}/\Gamma \ll 1$. For the probability of the two-photon generation, one derives the following result from Equations (5) and (6)

(8) $$\begin{array}{c} P_{2}\left(t\right)\propto \frac{U^{2}{\left|\alpha_{+}\left(0\right)\right|}^{4}}{\Gamma^{2}}{\left(1-\exp\left\{-\Gamma t\right\}\right)}^{2}. \end{array}$$

Equations (7) and (8) describe the action of the "built-in" pump rejection. The pump exponentially decays, whereas the probability of the pair creation approaches its maximal value for the propagation time when the pump is almost completely rejected. One can arbitrarily enhance pump rejection by simply increasing the propagation time.

Of course, the unavoidable presence of waveguide loss limits the possible extension of the waveguide, as it leads to the destruction of the generated photon pairs. However, our scheme has an additional intrinsic mechanism of pump rejection: spatial symmetry. A common 50/50 beamsplitter at the outcome of our device allows for the remnant of the driving field (which is in the symmetric mode) to be filtered out. A similar spatial-filtering mechanism was recently suggested for pump rejection for the SPDC-based waveguide source of photon pairs [16,17].

## 3. Results and Discussions

### 3.1. Operational Regime

Let us clarify the conditions of operation for our pump-rejecting pair generator. We want to have, at the output of the device, an average number of photon pairs that is much higher than the numbers of surviving pump photons and single photons appearing after destruction of the pairs by unavoidable realistic linear loss. The condition of our scheme functioning at some time *T* can be given in the following way

(9) $$\begin{array}{c} P_{2}\left(T\right)\gg \frac{1}{2}P_{1}\left(T\right),\frac{1}{2}{\left|\alpha_{+}\left(T\right)\right|}^{2}, \end{array}$$

where $P_{1}\left(T\right)$ is the probability of single-photon generation at the time *T*.

When one takes into account the linear loss γ and considers its rate, γ<<Γ, as being much lower than engineered loss, Γ, for a weak pump $\left(U|\alpha_{+}\left(0\right){|}^{2}/\Gamma \ll 1\right)$, the probability of the two photon generation can be derived from Equations (5)–(7), as:

(10) $$\begin{array}{c} P_{2}\left(t\right)\approx \frac{1}{8}\frac{U^{2}{\left|\alpha_{+}\left(0\right)\right|}^{4}}{\Gamma^{2}}\exp\left\{-2\gamma t\right\}{\left(1-\exp\left\{-\Gamma t\right\}\right)}^{2}. \end{array}$$

**Figure 2:**
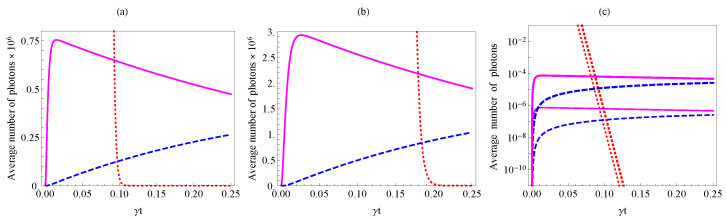
Average number of twin-photons (solid lines), single-photons (dashed lines), and pump photons (dotted lines) as given by Equations (7), (10) and (12). (**a**,**b**) correspond to Γ=400γ and Γ=200γ respectively and the initial average number of photons of the pump $|\alpha_{+}{\left(0\right)|}^{2}=10^{10}$. (**c**) corresponds to $|\alpha_{+}{\left(0\right)|}^{2}=10^{10}$ (thin lines) and $|\alpha_{+}{\left(0\right)|}^{2}=10^{11}$ (thick lines) and Γ=400γ. For all of the figures, the squared nonlinearity is $U^{2}=10^{-20}\gamma^{2}$.

Equations (7) and (10) show that, for the condition (9) to be fulfilled in the presence of linear loss, one needs the interaction to take place over a time interval that is larger than the time that maximizes the probability of pair generation. Indeed, from Equation (10), an estimate for the time of the maximal probability of obtaining a photon pair is given by:

(11) $$\begin{array}{c} t_{\max}\approx -\frac{1}{\Gamma }\ln\left(\frac{\gamma }{\Gamma }\right). \end{array}$$

one can see that the optimal time (11) does not yield a large pump rejection. For pump intensity, the degree of rejection is just the ratio of loss rates, $x=\frac{\gamma }{\Gamma }$. However, it is easy to see that *n*-time increase in the interaction time over $t_{max}$ drastically suppresses the pump (as $x^{n}$), but leads to relatively small decrease of two-photon generation probability. Figure 2 illustrates this situation for a rather large (∼$10^{10}$) initial number of pump photons. Notice that, for the illustrated case, about 150 dB suppression of the pump takes place.

The probability of single-photons as result of the pair decay can be derived from Equations (5)–(7), as

(12) $$\begin{array}{c} P_{1}\left(t\right)\approx \frac{1}{4}\frac{U^{2}{\left|\alpha_{+}\left(0\right)\right|}^{4}}{\Gamma^{2}}\exp\left\{-\gamma t\right\}\left(1-\exp\left\{-\gamma t\right\}-\frac{2\gamma }{\Gamma }\left(1-\exp\left\{-\Gamma t\right\}\right)+\frac{\gamma }{2\Gamma }\left(1-\exp\left\{-2\Gamma t\right\}\right)\right) \end{array}$$

so, one can see from Equations (7), (10) and (12) that to fulfill the condition (9), the interaction time *T* should satisfy

(13) $$\begin{array}{c} \frac{1}{\gamma }\gg T>\frac{1}{\Gamma }\ln\left(\frac{4\Gamma^{2}}{U^{2}{\left|\alpha_{+}\left(0\right)\right|}^{2}\delta }\right), \end{array}$$

where the parameter $\delta =|\alpha_{+}{\left(T\right)|}^{2}/2P_{2}\left(T\right)\ll 1$ defines the acceptable level of the pump rejection.

The condition (13) imposes a limit on the relation between the “natural” linear loss γ and the engineered one, Γ, for our scheme to be functional and to provide for generation of photon pairs with low admixture of single-photons. Generally, Γ≫γ is required. For the example that is shown in Figure 2a,b, the engineered loss rate should be at least two orders of magnitude larger than the natural loss rate to give a probability of photon pair generation much exceeding that of single-photons.

As we demonstrate later on, such a large engineered loss is completely feasible and it can be easily achieved while using different material platforms (for example, waveguides in fused silica or planar waveguides in InP). Of course, an increase in the engineered loss rate will lower the pair generation probability. However, this can be compensated by an increase in pump intensity. As it follows from the condition (13), even a large increase in pump intensity still does not lead to a significant increase of the interaction time that is required for the pump rejection. Figure 2c illustrates this situation: a tenfold increase in pump intensity leads to a hundredfold increase of $P_{1,2}$, but only few percent change in the required interaction time (see Equations (10) and (12) ).

### 3.2. Quantum Perturbation Theory

In this Section, we confirm that theresults of the previous section, obtained in the approximation of a coherent pump, remain valid, even beyond this approximation, i.e., when both modes are treated using quantum perturbation theory. These results continue to hold provided that the weak pump approximation remains valid, $\lambda =U|\alpha_{+}{\left(0\right)|}^{2}/\Gamma \ll 1$. Using a perturbation theory for the operators, in Appendix A the following expressions are derived for the average numbers of photons in each collective mode:

(14) $$\begin{array}{cc} \langle n_{+}\left(t\right)\rangle & =|\alpha_{+}{\left(0\right)|}^{2}e^{-(\Gamma +\gamma )t}+O\left(\lambda^{2}\right),\\ \langle n_{-}\left(t\right)\rangle & =\frac{U^{2}{\left|\alpha_{+}\left(0\right)\right|}^{4}}{2\Gamma (2\Gamma +\gamma )}\frac{e^{-2(\Gamma +\gamma )t}}{\Gamma +\gamma }\times \left\{\eta (t)\Gamma +\gamma \zeta (t)\right\}+O\left(\lambda^{3}\right), \end{array}$$

with functions $\eta \left(t\right)=e^{(2\Gamma +\gamma )t}-2e^{\Gamma t}+1$ and $\zeta \left(t\right)=1-e^{\Gamma t}$.

The average number of pump photons $\langle n_{+}\left(t\right)\rangle$ agrees with Equation (7) that was obtained by the semiclassical approximation. Moreover, a higher-order correction that is given by Equation (A14) shows that, even when the average number of the pump photons is comparable with the average number of generated photons, the deviation from the semiclassical formula Equation (7) is only small.

We also check that the result (14) for the average number of photons in the antisymmetric mode, $\langle n_{-}\left(t\right)\rangle$, for γ≪Γ corresponds to the sum $2P_{2}\left(t\right)+P_{1}\left(t\right)$, as given by Equations (10) and (12).

Thus, we deduce that, in the limit of the weak pump, $U|\alpha_{+}{\left(0\right)|}^{2}/\Gamma \ll 1$, semiclassical pump approximation gives results that are very close with the quantum analysis up to the very low numbers of photons.

### 3.3. Realizations

**Figure 3:**
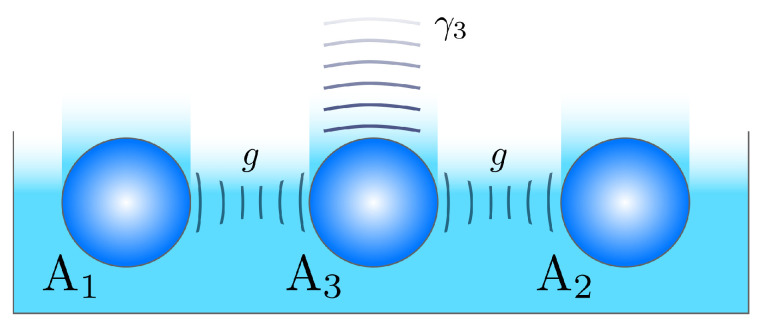
An example of three-waveguide realization of the basic Scheme (1). The waveguide $A_{3}$ is subjected to engineered loss with the rate $\gamma_{3}$. Both waveguides $A_{1}$ and $A_{2}$ are unitary coupled to the waveguide $A_{3}$; the coupling constant is *g*.

The simplest way to realize the collective loss that is described in the Scheme (1) is to couple two single-mode waveguides to a third lossy waveguide (see Figure 3). Subsequently, for strong loss in the middle waveguide, one can adiabatically exclude the third mode ($A_{3}$ in Figure 3) and arrive at the master Equation (1). Under symmetric coherent excitation of both waveguides $A_{1,2}$, the regime of the pair generation can be realized. This scheme has been suggested for the realization of different kinds of nonlinear loss [29,32], and for dissipative beamsplitting/equalization [30]. The adiabatic elimination was discussed in detail in the recent work [31]. Basically, if the side single-mode waveguides are coupled to the central one with the coupling rates *g*, and the central waveguide is subjected to the linear loss with the rate $\gamma_{3}$, the collective loss rate is

(15) $$\Gamma \approx 8\frac{g^{2}}{\gamma_{3}}.$$

Equation (15) shows that ratios of the engineered and “natural” loss rates given by condition (13) are easily feasible in three-waveguide structures that are depicted in Figure 3. For example, in the recent works [30,31], an all-glass scheme with laser-inscribed single-mode waveguides was realized with the strong linear loss in the third waveguide induced by a long “tail” of coupled waveguides. In such "tailed" glass structures, one can routinely have *g* of about 200–300 m${}^{-1}$ in the infrared-visible wavelength regions, and a rate $\gamma_{3}$ of about four times their value, allowing for Γ of about 400–600 m${}^{-1}$. Even for highly nonlinear glass, such as chalcogenide glass IG2, with losses about 12 m${}^{-1}$ at 1 μm wavelength, the condition (13) can be easily satisfied. For common silica glass, propagation loss can be less than 2 m${}^{-1}$ in the infrared and optical wavelength regions [33,34]. Even better ratios are possible while using drawing techniques for waveguide structures (which is commonly used for producing photonic crystal fibers [35]). There, the propagation loss can be almost as low as for the conventional index-guiding optical single-mode fibers, and be lower than $10^{-4}$ m${}^{-1}$ [36].

**Figure 4:**
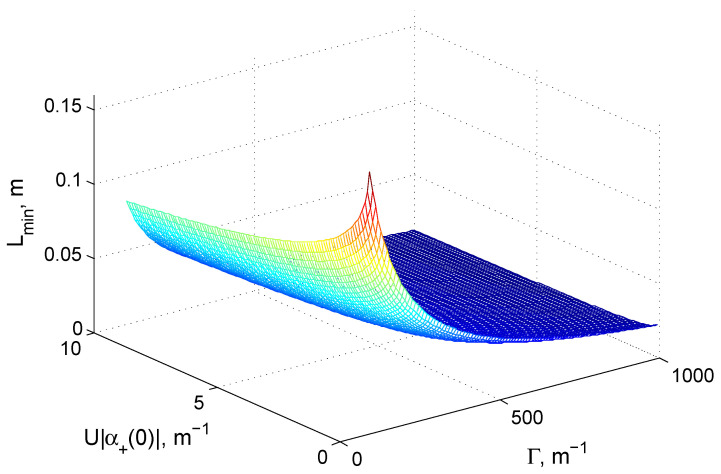
A minimal waveguide length, $L_{min}$, providing for pump rejection with δ=0.1 for typical parameters of the glass waveguides and weak pump.

Obviously, all-glass structures might face size problems when integrated into optical circuits. For example, while the width of the structure for 800 nm wavelength will be less than 50 μm, the height of the “tail” (which should be of more that 10 waveguides [30,31]) might be 0.5 mm and more. More serious is the problem with the necessary propagation length. Figure 4 shows the minimal interaction length, $L_{min}$, providing for pump rejection with δ=0.1, as given by the left-hand side of the condition (13) and for the typical system parameters that are discussed in this Section. One can see that, for quite a wide range of parameters, the minimal length that is required to reduce the average number of residual pump photons much below the number of generated photons, is of about few cm. This limits the possibilities of integration.

However, one can strongly reduce the required device length by combining the pump self-rejection and spatial rejection scheme: the residual pump can be rejected by 50/50 beamsplitter at the output of the device. Indeed, increasing the coupling strength in order to provide an order of magnitude larger Γ than those that are typical in all-glass structures, one can achieve more than 50 dB pump self-rejection with a structure of just few millimeter length. This approach seems to be useful with planar ridge waveguides built on such common platforms as silicon [37] or indium phosphide-(InP) [38]-based planar semiconductor structures. These can provide for a possibility to have much higher coupling (and, correspondingly, Γ) than in glass, at the price of having much larger propagation loss (for example, of about 50–100 m${}^{-1}$ for InP waveguides [38] or silicon waweguides [37]).

Of course, an increase in Γ leads to a decrease in the pair generation rate. However, the larger nonlinearity and tighter mode confinement more than compensate for it. Indeed, for the device length larger than $\Gamma^{-1}$, from Equation (10), the pair generation rate is given by [31]

(16) $$\begin{array}{c} R_{2}\approx {\left(\frac{2\pi n_{2}}{\lambda S}P_{w}\right)}^{2}\frac{c}{8\Gamma }\eta , \end{array}$$

where $P_{w}$ is the input power, *c* is the speed of light, λ is the wavelength, *S* is the waveguide modal area, $n_{2}$ is the nonlinear refractive index, and $\eta =e^{-2\gamma T}$ is extinction coefficient describing $P_{2}\left(T\right)$ reduction by single-photon loss.

For fused silica waveguides with typical values of $n_{2}\approx 2\times 10^{-20}\;$m${}^{2}$W${}^{-1}$, for g=300m${}^{-1}$ and negligibly small linear loss, at λ=800nm for a single-mode waveguide with typical modal area of $5\times 10^{-11}\mu$m${}^{2}$, about 180 W input power is needed in order to reach of about 1 KHz pair generation rate. This power value can be lowered by implementing weaker waveguide coupling for low-loss long fiber-like structures. However, as Equation (16) shows, it is much more advantageous to take a material with higher nonlinearity and tighter field localization, than to aim for lower Γ and longer structures.

For IG2-glass waveguides with the same modal area and $n_{2}$ of about two orders of magnitude larger than for fused silica, it is sufficient to have less than 200μW for reaching the same generation rate. With a single-mode silicon waveguides, one can have much tighter mode localization (for example, S≈5μm${}^{2}$ at 1550 nm wavelength [39]), and much higher non-linearity. For example, for such a silicon waveguide with a large Γ=1000m${}^{-1}$ at the wavelength of 1550 nm, just few μW input power suffice for reaching the 1 KHz generation rate [40].

Thus, the highly nonlinear planar waveguide structure that is schematically shown in Figure 3 is a prospective platform for realizing bright integrated generators of photon pairs with efficient pump self-rejection.

Further, our generator is robust with respect to the Raman scattering noise that commonly arises in photon pair generators implementing SFWM [41]. Such noise manifests as uncorrelated photons in waveguides and they can be the dominant source of noise in SFWM pair-generating devices [42]. However, in our devices, the Raman scattering produces uncorrelated photons in the symmetric mode, which is subject to strong engineered loss.

Finally, we notice that imperfections in the waveguide structure leading to the asymmetric coupling between waveguides might negatively affect the discussed scheme. Asymmetry will lead to the generation of uncorrelated single photons in the asymmetric mode [29]. However, as long as the discrepancy in coupling constants is not large in comparison with their values, the effect is negligible. More precisely, if the first waveguide couples to the dissipative waveguide with the rate g+δ>0, and the second waveguide couples to the dissipative waveguide with the rate g−δ>0, the effect of asymmetry will be negligible for g≫4|δ| [29]. In practice, it means that the deviations in distance between waveguides should be much lower than this distance, which is perfectly feasible [30].

### 3.4. Extention to NOON States

**Figure 5:**
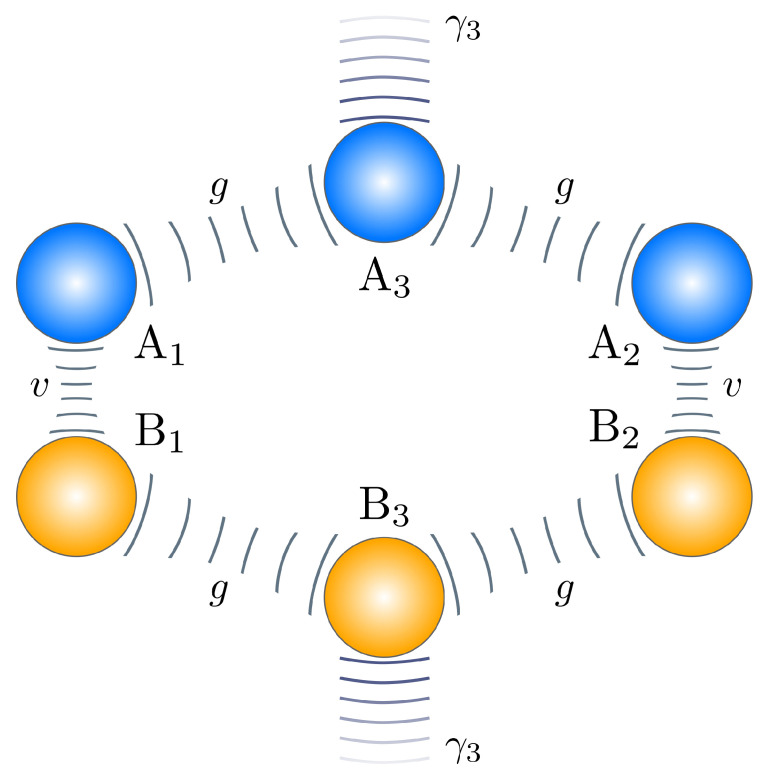
An example of six-waveguide realization of the NOON state generation scheme. The depicted structure shows two mirrored three-waveguide structures of Figure 3 with waveguides labeled as $A_{j}$ for the upper, and $B_{j}$ for the lower structures. The waveguides $A_{3}$ and $B_{3}$ are subject to strong engeneered loss. The waveguide $A_{1}$ is unitary coupled with the waveguide $B_{1}$, the waveguide $A_{2}$ is unitary coupled with the waveguide $B_{2}$; the coupling constant is *v*. Other parameters are shown in Figure 3.

**Figure 6:**
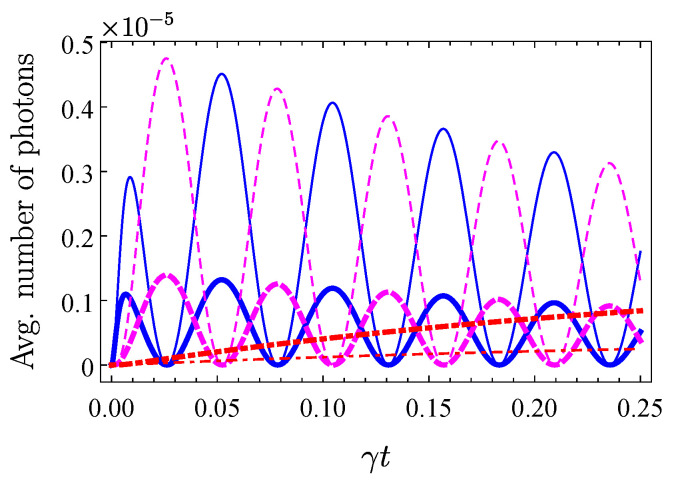
Average number of photons corresponding to the generation of two photons in one of the antisymmetric modes (blue solid lines) and of two photons being simultaneously in both antisymmetric modes (magenta dashed lines) according to Equation (17) (they are, respectively, $2\times P_{2,0}$ and $P_{1,1}$). Red dashed-dotted lines show the probability of finding a single photon only in one of the antisymmetric modes, due to photon loss from one of the previous configurations. The coupling constant has been chosen to be v=30γ. Thick lines correspond to Γ=400γ, thin lines correspond to Γ=200γ. Other parameters are the same as in Figure 2.

The described principle of the pump self-rejection can also be applied for more complicated setups than just the two dissipatively symmetrically coupled waveguides considered so far in this work. Any coherent diffusive photonic circuit working by collective loss (see Ref. [30] for several examples of such devices) can be implemented for the pump self-rejection scheme, as it is described above. As an example, let us consider a scheme that allows for us to simultaneously produce single-photon states in different spatial modes. Two-photon NOON states may then be generated by interfering these photons. The setup allowing this, Figure 5, consists of two mirror-imaged two-mode devices considered before. The state-carrying waveguides are unitarily coupled in the usual way, while the modes $A_{1}$, $B_{1}$, and $A_{2}$, $B_{2}$ are additionally coupled with rate *v*. This single-photon exchange process is described by the standard Hamiltonian, $W=\hbar v(a_{1}^{†}b_{1}+a_{2}^{†}b_{2}+h.c.)$, where *v* is the coupling rate, the operators $a_{j}$ describe state-carrying modes of the upper half of the device, and operators $b_{j}$ describe modes of the lower half of the device (the whole scheme is considered in detail in Appendix B). It is easy to see that, under Hamiltonian *W*, only the modes with the same symmetry are coupled. If one puts into all four state-carrying modes ($A_{1,2}$, $B_{1,2}$) coherent states with the same amplitude (say, α), then in the limits of low “natural” loss, low pump intensity and large interaction time T≫1/Γ, the photon-number probabilities read

(17) $$\begin{array}{c} P_{1,1}\propto \frac{U^{2}{\left|\alpha \left(0\right)\right|}^{4}}{\Gamma^{2}}{\left(\sin\left\{2vT\right\}\right)}^{2},\\ P_{2,0}\propto \frac{U^{2}{\left|\alpha \left(0\right)\right|}^{4}}{\Gamma^{2}}{\left(\cos\left\{2vT\right\}\right)}^{2} \end{array}$$

probability $P_{1,1}\left(T\right)$ denotes the probability to simultaneously generate two photons, one in each of the antisymmetric superposition modes, while $P_{2,0}\left(T\right)$ denotes the probability to generate two photons in just one of the antisymmetric modes. Equation (17) show that, for any designed device parameters, one can always choose the coupling constant *v* (for example, just by adjusting the distance between the upper and lower parts of the device), as to have a photon in each antisymmetric mode at the output, and produce two-photon NOON state by interfering these photons. Figure 6 visualizes the relevant probabilities, where we include the effects of linear loss.

## 4. Conclusions

We have suggested and discussed a scheme of pump self-rejection for the generation of photon pairs and two-photon path-entangled states by the four-wave mixing process in the system of Kerr nonlinear waveguides. The cornerstone of our scheme is the strong collective loss acting on the waveguides pairs. This loss strongly affects the symmetric collective modes, but leaves the antisymmetric mode intact. By driving the symmetric collective mode, we obtain photon pairs in the antisymmetric mode. For a sufficiently long device, the pump is completely filtered out, but photon loss and the appearance of uncorrelated single photons limit the length.

We have analyzed device design and performance for the realistic waveguide structures in both glass and for such platforms as indium phosphide (InP) and silicon based planar semiconductor structures. We have demonstrated that the available nonlinearities, waveguide coupling rates, and “natural” linear loss will allow for the fabrication of functional, high-quality photon-pair generators with pump self-rejection. In addition, one can combine self-rejection with the spatial rejection: it is possible to filter out the remnant of the pump just by interfering the output modes and in order to separate the antisymmetric mode.

We have also demonstrated the way to use our pump self-rejection for more complicated photonic circuits for the generation of entangled photons. Duplicating our two-waveguide set-up, it is possible to simultaneously produce two photons in two different group of waveguides, and generate NOON states.

## Appendix A. Heisenberg-Picture Operator Perturbation Expansion for the Photon Number

To check the validity of the predictions obtained by the simplest scheme, and in order to investigate the dynamical regime in which the results start to deviate from those obtained from the basic assumptions made in Section 2, the two-mode description of our photon source is explored. To do this, we consider the two mode master equation Equation (2), which is expressed in terms of the collective modes.

One could solve directly the master equation to obtain the time evolution of the density matrix and subsequently use the properties of the trace to obtain the observable quantities. However, an important simplification of the problem is achieved if we consider the evolution of the operators in the Heisenberg picture [43]. Thus, we start with the adjoint master equation:

(A1) $$\begin{array}{c} \frac{d}{dt}A(t)=i\left[H+V,A\left(t\right)\right]+\left(\Gamma +\gamma \right)L^{†}\left(a_{+}\right)A(t)+\gamma L^{†}\left(a_{-}\right)A(t), \end{array}$$

for any operator *A*. Since the Hamiltonian of Equation (3) is diagonal in the Fock state basis, it does not manifest itself when we calculate the probability of photon pair generation, nor when we calculate the expected value for the photon number in each collective mode. This way we can focus our attention in the interaction Hamiltonian Equation (4) describing the conversion of two-photons from the symmetric mode into the antisymmetric one, and vice versa.

The presence of the non-linear Hamiltonian precludes an exact solution of Equation (A1). However, in the conditions previously mentioned $\lambda =U|\alpha_{+}{\left(0\right)|}^{2}/\Gamma$ is a small parameter, and therefore we can obtain approximate solutions for the evolution of the operator *A* in the form of a power series in the small parameter, where we require the interaction Hamiltonian V=O(λ).

We consider a perturbation expansion of the photon number operator:

(A2) $$\begin{array}{c} \begin{array}{c} \left(a_{\pm }^{†}a_{\pm }\right)\left(t\right)={\left(a_{\pm }^{†}a_{\pm }\right)}^{\left(0\right)}\left(t\right)+{\left(a_{\pm }^{†}a_{\pm }\right)}^{\left(1\right)}\left(t\right)+{\left(a_{\pm }^{†}a_{\pm }\right)}^{\left(2\right)}\left(t\right)+\cdots \end{array} \end{array}$$

where the *j*-th term of the expansion is proportional to $\lambda^{j}$, i.e., ${\left(a_{\pm }^{†}a_{\pm }\right)}^{\left(j\right)}=O\left(\lambda^{j}\right)$

We can solve Equation (A1) for $a_{\pm }^{†}a_{\pm }$ by solving an equivalent set of equations which are found by identifying the terms of the same order in λ. These are equations on ${\left(a_{\pm }^{†}a_{\pm }\right)}^{\left(j\right)}$ that can be solved once we know the solution for ${\left(a_{\pm }^{†}a_{\pm }\right)}^{\left(j-1\right)}$. In particular, we have:

(A3) $$\begin{array}{cc} \frac{d}{dt}{\left(a_{\pm }^{†}a_{\pm }\right)}^{\left(0\right)}\left(t\right) & =\Gamma_{-}L^{†}\left(a_{-}\right){\left(a_{\pm }^{†}a_{n}\right)}^{\left(0\right)}\left(t\right)+\Gamma_{+}L^{†}\left(a_{+}\right){\left(a_{\pm }^{†}a_{\pm }\right)}^{\left(0\right)}\left(t\right), \end{array}$$

(A4) $$\begin{array}{cc} \frac{d}{dt}{\left(a_{\pm }^{†}a_{\pm }\right)}^{\left(j\right)}\left(t\right) & =i\left[V,{\left(a_{\pm }^{†}a_{\pm }\right)}^{\left(j-1\right)}\left(t\right)\right]+\Gamma_{-}L^{†}\left(a_{-}\right){\left(a_{\pm }^{†}a_{\pm }\right)}^{\left(j\right)}\left(t\right)+\Gamma_{+}L^{†}\left(a_{+}\right){\left(a_{\pm }^{†}a_{\pm }\right)}^{\left(j\right)}\left(t\right) \end{array}$$

with $\Gamma_{+}=\Gamma +\gamma$ and $\Gamma_{-}=\gamma$. The zero-th order Equation (A3) can be readily solved as it does not contain the Hamiltonian. As the initial condition, we require that the Heisenberg and Schrödinger operators coincide at t=0. We understand in what follows that the operators without explicit time dependence are Schrödinger operators. We reach the zero-th order solution:

(A5) $$\begin{array}{cc} {\left(a_{\pm }^{†}a_{\pm }\right)}^{\left(0\right)}\left(t\right) & =a_{\pm }^{†}a_{\pm }e^{-\Gamma_{\pm }t} \end{array}$$

Photons in the symmetric and antisymmetric modes decay exponentially at rates Γ+γ and γ respectively. The knowledge of the zero-th order solution allows us to solve for the first order:

(A6) $$\begin{array}{c} \begin{array}{c} {\left(a_{\pm }^{†}a_{\pm }\right)}^{\left(1\right)}\left(t\right)=\frac{iUe^{-(\Gamma +2\gamma )t}\left(e^{\Gamma_{\mp }t}-1\right)}{2\Gamma_{\mp }}\left(a_{\pm }^{†}a_{\pm }^{†}a_{\mp }a_{\mp }-a_{\mp }^{†}a_{\mp }^{†}a_{\pm }a_{\pm }\right) \end{array} \end{array}$$

Similarly, we obtain the second order solution:

(A7) $$\begin{array}{c} \begin{array}{c} {\left(a_{\pm }^{†}a_{\pm }\right)}^{\left(2\right)}\left(t\right)=f_{1\pm }^{\left(2\right)}\left(t\right){\left(a_{\pm }^{†}\right)}^{2}a_{\pm }^{2}+f_{2\pm }^{\left(2\right)}\left(t\right){\left(a_{\mp }^{†}\right)}^{2}a_{\mp }^{2}+f_{3\pm }^{\left(2\right)}{\left(a_{\pm }^{†}\right)}^{2}a_{\pm }^{2}\left(a_{\mp }^{†}\right)a_{\mp }+f_{4\pm }^{\left(2\right)}\left(t\right)\left(a_{\pm }^{†}\right)a_{\pm }{\left(a_{\mp }^{†}\right)}^{2}a_{\mp }^{2} \end{array} \end{array}$$

where $f_{i\pm }^{\left(2\right)}\left(t\right)$ with i=1,⋯4 are functions of time that determine the dynamical evolution at second order. The explicit expression for these functions can be obtained after substitution of the expressions Equations (A6) and (A7) in Equation (A4). The result is a differential equation for each of these functions that we obtain by identifying the coefficients of the corresponding operators. After integration, we obtain:

(A8) $$\begin{array}{cc} f_{1\pm }^{\left(2\right)}\left(t\right)= & \pm \frac{U^{2}e^{-(\Gamma +2\gamma )t}}{2\Gamma \Gamma_{\mp }\left(\pm \Gamma +\Gamma_{\mp }\right)}\left[\Gamma_{\mp }\left(1-e^{\mp \Gamma t}\right)\pm \Gamma \left(1-e^{\Gamma_{\mp }t}\right)\right], \end{array}$$

(A9) $$\begin{array}{cc} f_{2\pm }^{\left(2\right)}\left(t\right)= & \mp \frac{U^{2}e^{-(\Gamma +2\gamma )t}}{2\Gamma \Gamma_{\mp }\left(3\Gamma_{\mp }-2\gamma -\Gamma \right)}\left[\left(2\gamma +\Gamma \right)\left(1-e^{\Gamma_{\mp }t}\right)+\Gamma_{\mp }\left(2e^{\Gamma_{\mp }t}+e^{\pm \Gamma t}-3\right)\right], \end{array}$$

(A10) $$\begin{array}{cc} f_{3\pm }^{\left(2\right)}\left(t\right)= & -\frac{U^{2}e^{-(2\Gamma_{\pm }+\Gamma_{\mp })t}}{\gamma (\Gamma +\gamma )(\Gamma +2\gamma )}\left[\Gamma_{\mp }+\Gamma_{\pm }e^{(\Gamma +2\gamma )t}-\left(\Gamma +2\gamma \right)e^{\Gamma_{\pm }t}\right], \end{array}$$

(A11) $$\begin{array}{cc} f_{4\pm }^{\left(2\right)}\left(t\right)= & \frac{U^{2}e^{-(2\Gamma_{\mp }+\Gamma_{\pm })t}}{2\Gamma_{\mp }^{2}}{\left(e^{\gamma_{\mp }t}-1\right)}^{2} \end{array}$$

the photons-number statistics that characterize our source of photon pairs can be obtained from the analytic expressions we have now derived. We assume that the symmetric mode is initially excited in a coherent state. The average values in the Heisenberg picture are obtained as $\langle A\left(t\right)\rangle =\mathrm{Tr}\left[A\left(t\right)\rho_{0}\right]$ with $\rho_{0}=|\alpha_{+}\left(0\right)\rangle \langle \alpha_{+}\left(0\right)|⊗|0\rangle \langle 0|$. For the symmetric mode:

(A12) $$\begin{array}{cc} \langle n_{+}\left(t\right)\rangle = & \langle n_{+}^{\left(0\right)}\left(t\right)\rangle +\langle n_{+}^{\left(2\right)}\left(t\right)\rangle +O\left(\lambda^{3}\right), \end{array}$$

(A13) $$\begin{array}{cc} \langle n_{+}^{\left(0\right)}\left(t\right)\rangle = & |\alpha_{+}{\left(0\right)|}^{2}e^{-(\Gamma +\gamma )t}, \end{array}$$

(A14) $$\begin{array}{cc} \langle n_{+}^{\left(2\right)}\left(t\right)\rangle = & -\frac{U^{2}{\left|\alpha_{+}\left(0\right)\right|}^{4}}{2\Gamma \gamma (\Gamma +\gamma )}e^{-2(\Gamma +\gamma )t}\left(\gamma +\Gamma e^{(\Gamma +\gamma )t}-\left(\Gamma +\gamma \right)e^{\Gamma t}\right), \end{array}$$

for the antisymmetric mode:

(A15) $$\begin{array}{cc} \langle n_{-}\left(t\right)\rangle & =\langle n_{-}^{\left(2\right)}\left(t\right)\rangle +O\left(\lambda^{3}\right), \end{array}$$

(A16) $$\begin{array}{cc} \langle n_{-}^{\left(2\right)}\left(t\right)\rangle & =\frac{U^{2}{\left|\alpha_{+}\left(0\right)\right|}^{4}}{2\Gamma (2\Gamma +\gamma )}\frac{e^{-2(\Gamma +\gamma )t}}{\Gamma +\gamma }\left(\eta (t)\Gamma +\gamma \zeta (t)\right), \end{array}$$

with functions $\eta \left(t\right)=e^{(2\Gamma +\gamma )t}-2e^{\Gamma t}+1$ and $\zeta \left(t\right)=1-e^{\Gamma t}$.

## Appendix B. NOON State Generator

The pair-generator scheme considered until now in this work is produces pairs of photons in the same (antisymmetric) mode. However, a simple modification of the scheme allows us to create photons in two different superposition modes, and thus to generate two-photon NOON states. This modification is two three-waveguide structures, as of Figure 3, with unitary coupling between the state-carrying modes depicted in Figure 5. After elimination of the modes $a_{3}$ and $b_{3}$, this system is described by the following master equation

(A17) $$\begin{array}{c} \frac{d}{dt}\rho =\sum_{x,j}\left(-i\frac{U}{2}\left[{\left(x_{j}^{†}\right)}^{2}x_{j}^{2},\rho \right]+\gamma L\left(x_{j}\right)\rho \right)-iv\left[a_{1}^{†}b_{1}+a_{2}^{†}b_{2}+b_{1}^{†}a_{1}+b_{2}^{†}a_{2},\rho \right]+\\ \frac{1}{2}\Gamma \left(L\left(a_{1}+a_{2}\right)+L\left(b_{1}+b_{2}\right)\right)\rho , \end{array}$$

where x=a,b, and $a_{j}$ denote the modes of upper half of the device, and $b_{j}$ denote modes of the lower half of the device. The constant *v* describes the coupling between the waveguides of the both halves.

After transforming to the basis $x_{\pm }=\frac{1}{\sqrt{2}}\left(x_{1}\pm x_{2}\right)$, Equation (A17) becomes

(A18) $$\begin{array}{c} \frac{d}{dt}\bar{\rho }=-\sum_{x=a,b}i\left[H_{x}+V_{x},\bar{\rho }\right]-iv\left[\left(a_{+}^{†}b_{+}+a_{-}^{†}b_{-}\right)+h.c.\right),\rho ]+\sum_{x=a,b}\left(\left(\Gamma +\gamma \right)L\left(x_{+}\right)+\gamma L\left(x_{-}\right)\right)\bar{\rho }, \end{array}$$

where the Kerr interaction Hamiltonian is

(A19) $$\begin{array}{c} H=\frac{U}{4}\left(n_{x+}^{2}+n_{x-}^{2}+4n_{x+}n_{x-}-n_{x+}-n_{x-}\right), \end{array}$$

and the two-photon exchange Hamiltonian is

(A20) $$\begin{array}{c} V_{x}=\frac{U}{4}\left({\left(x_{+}^{†}\right)}^{2}x_{-}^{2}+h.c.\right) \end{array}$$

the operators $n_{x\pm }=x_{\pm }^{†}x_{\pm }$ are photon-number operators for the superposition modes. Notice that the photon exchange between symmetric and antisymmetric modes in the scheme described by Equations (A18)–(A20) occurs only through the two-photon exchange (A20).

In the interaction picture with respect to the unitary coupling Hamiltonian $W=v[a_{-}^{†}b_{-}+h.c.]$, considering the states of symmetric modes to be classical, after averaging over the symmetric modes one obtains

(A21) $$\begin{array}{c} \frac{d}{dt}\varrho \approx -i\left[V\left(t\right),\varrho \right]+\gamma \left(L\left(a_{-}\right)+L\left(b_{-}\right)\right)\varrho , \end{array}$$

where we have assumed both weak nonlinearity and weak pump. The Hamiltonian:

(A22) $$\begin{array}{c} V\left(t\right)=\frac{U}{4}\sum_{x=a,b}(n_{x-}^{2}+n_{x-}\left(4\right|\alpha_{+}{\left(t\right)|}^{2}-1))+\frac{U}{4}(\alpha^{2}\left(t\right)\left[{\left(a_{-}^{†}\right)}^{2}+{\left(b_{-}^{†}\right)}^{2}\right]\cos\left\{2vt\right\}+\\ 2ia_{-}^{†}b_{-}^{†}\sin\left\{2vt\right\}+h.c.) \end{array}$$

with

(A23) $$\begin{array}{c} \alpha \left(t\right)\approx \alpha \left(0\right)\exp\{-\frac{1}{2}\left(\Gamma +\gamma \right)t-ivt\}, \end{array}$$

where α(0) is the initial amplitude of both the symmetric modes $a_{+}$ and $b_{+}$.

As it is with the simpler version of the scheme, Equation (A23) describes the exponential pump rejection.

To show how the NOON states generation functions, let us neglect an influence of the “natural loss”. Then, for large propagation time T≫1/Γ, and for the unitary exchange rate much less than the engineered loss rate, |v|≪Γ, after returning to the original picture with respect to the unitary exchange operator *W*, one obtains the following result for the probability of two photons being simultaneously in both antisymmetric superposition modes

$$\begin{array}{c} P_{1,1}\propto \frac{U^{2}{\left|\alpha \left(0\right)\right|}^{4}}{\Gamma^{2}}{\left(\sin\left\{2vT\right\}\right)}^{2} \end{array}$$

and the probability to have two photons in one of the antisymmetric modes

$$\begin{array}{c} P_{2,0}\propto \frac{U^{2}{\left|\alpha \left(0\right)\right|}^{4}}{\Gamma^{2}}{\left(\cos\left\{2vT\right\}\right)}^{2} \end{array}$$

interfering these two photons on the 50/50 beamsplitter, one gets the 2-photon NOON state.
